# Transcription Factor ATF4 Induces NLRP1 Inflammasome Expression during Endoplasmic Reticulum Stress

**DOI:** 10.1371/journal.pone.0130635

**Published:** 2015-06-18

**Authors:** Andrea D’Osualdo, Veronica G. Anania, Kebing Yu, Jennie R. Lill, Randal J. Kaufman, Shu-ichi Matsuzawa, John C. Reed

**Affiliations:** 1 Cell Death and Survival Networks Program, Sanford-Burnham Medical Research Institute, La Jolla, California, United States of America; 2 Department of Protein Chemistry, Genentech Inc, South San Francisco, California, United States of America; 3 Degenerative Diseases Research Program, Sanford-Burnham Medical Research Institute, La Jolla, California, United States of America; 4 Pharma Research and Early Development (pRED), Roche, Basel, Switzerland; IISER-TVM, INDIA

## Abstract

Perturbation of endoplasmic reticulum (ER) homeostasis triggers the ER stress response (also known as Unfolded Protein Response), a hallmark of many pathological disorders. However the connection between ER stress and inflammation remains largely unexplored. Recent data suggest that ER stress controls the activity of inflammasomes, key signaling platforms that mediate innate immune responses. Here we report that expression of NLRP1, a core inflammasome component, is specifically up-regulated during severe ER stress conditions in human cell lines. Both IRE1α and PERK, but not the ATF6 pathway, modulate *NLRP1* gene expression. Furthermore, using mutagenesis, chromatin immunoprecipitation and CRISPR-Cas9-mediated genome editing technology, we demonstrate that ATF4 transcription factor directly binds to *NLRP1* promoter during ER stress. Although involved in different types of inflammatory responses, XBP-1 splicing was not required for *NLRP1* induction. This study provides further evidence that links ER stress with innate

## Introduction

The endoplasmic reticulum (ER) is a specialized organelle that controls the biogenesis of membrane-anchored and secreted proteins[1]. Beyond this biosynthetic role, the ER is also a dynamic cellular compartment that plays critical roles in calcium storage[2] and cellular homeostasis[3]. Perturbations in the ER triggered by increased protein synthesis, accumulation of misfolded proteins, nutrient deprivation, or alterations in the calcium or redox balance can lead to a condition called ER stress[4, 5]. ER imbalance activates a transcriptional and translational program known as the unfolded protein response (UPR), an adaptive signaling cascade that primarily acts to restore ER homeostasis[6]. Specifically, the UPR activates three main signaling pathways initiated by different ER transmembrane sensors: PERK, IRE1α and ATF6, which cause initial shutdown in mRNA translation along with up-regulation of genes encoding protein chaperones and ER-associated degradation (ERAD) machinery[7, 8]. However, if ER stress is not mitigated, a cell death program is then initiated[9, 10].

In addition to its adaptive role, the UPR also regulates the inflammatory state, a circumstance that is frequently present in many metabolic conditions such as obesity and diabetes[11], as well as neurodegenerative diseases[12] and a variety of bacterial and viral infections[13–15]. The mechanisms linking ER stress to inflammation are only partly understood (reviewed in [16]).

The pleiotropic transcription factor XBP-1s, generated through unconventional mRNA splicing mediated by IRE1α, enhances protein folding and has been shown to control intestinal inflammation in patients affected with inflammatory bowel disease[17]. IRE1α-XBP-1s signaling is also activated by extracellular Toll-like receptors (TLRs) during bacterial infections and is responsible for sustained pro-inflammatory cytokine secretion by macrophages[18]. In addition to XBP-1 activation, IRE1α controls inflammatory cytokine secretion in rheumatoid arthritis through the adaptor molecule TRAF6[19] and leads to NF-κB and JNK activation pathways by recruiting TRAF2, an adapter protein and E3 ligase associated with several TNF-family receptors[20]. Moreover, PKR-like ER-resident kinase (PERK)-mediated phosphorylation of the alpha subunit of eukaryotic translation initiation factor eIF2α also contributes to NF-κB activation by repressing the synthesis of the inhibitor IkBα[21]. By means of eIF2α phosphorylation, PERK promotes translation of ATF4 mRNA, a transcription factor that is a central hub of the more general integrated stress response (ISR)[22] and which has been recently implicated in viral infections, Alzheimer’s disease and diabetes[23, 24].

Members of the NLR (Nucleotide-binding NACHT domain and Leucine-Rich Repeat containing proteins) family of proteins are critical components of host innate immunity in many species[25]. Interestingly, IRE1α and PERK pathways have recently been shown to regulate NLRP3 inflammasome-dependent cell death and IL-1β secretion through the thioredoxin-interacting protein TXNIP[26, 27]. Another NLR protein, NLRP1, has also been shown to participate in both cell death[28] and IL-1β secretion[29], but its involvement in UPR signaling has not previously been reported. In this study, we investigated the regulation of NLRP1 inflammasome in response to ER perturbations. We found that both PERK and IRE1α stimulate *NLRP1* gene transcription through the transcription factor ATF4, involving a mechanism that is independent of XBP-1 mRNA splicing.

## Materials and Methods

### Cell culture

Cell lines were cultured at 37°C with 5% CO_2_. THP-1, K562 and Jurkat cells were maintained in RPMI-1640 medium, HeLa cells in Dulbecco’s modified Eagle’s medium (DMEM) and HCT116 cells in McCoy’s-5A medium. All media (Corning-Cellgro) were supplemented with 10% heat-inactivated fetal bovine serum (Sigma) and penicillin-streptomycin (100IU/ml and 100μg/ml, respectively; Corning-Cellgro). All cell lines were obtained from ATCC.

### Reagents, plasmids, and antibodies

DMSO (472301), tunicamycin (T7765), thapsigargin (T9033), brefeldin A (B7651), actinomycin D (A1410), DRB (D1916) and Forskolin (F6886) were purchased from Sigma. Monosodium urate crystals were prepared from uric acid (U0881, Sigma) as previously described[30]. PolyI:C, flagellin, MDP and R837 were from Invivogen. All siRNA were purchased from Ambion. Different shRNA were cloned in pLKO.1 (Addgene #10878) and/or in Tet-pLKO-puro (Addgene #21915) vectors as previously described[31]. A complete list of siRNA and shRNA used is presented in S1 Table. IRE1α (#3294) and PERK (#3192) antibodies were from Cell Signaling Technology, ATF6 antibody (ab122897) was from Abcam, and XBP-1s antibody (clone 143F) was from Biolegend. ATF4 antibody for ChIP (sc-22800) was from Santa Cruz. NLRP1 antibody (AF6788) was from R&D Systems.

### RNA extraction, semiquantitative RT-PCR and real-time qPCR

After ER stress induction, cells were washed twice with PBS and total RNA extraction was performed using an RNeasy kit (Qiagen). RNA concentration and purity was measured using a NanoDrop spectrophotometer (Thermo Scientific). A total of 2μg RNA was retro-transcribed using SuperScript III reverse transcriptase (Life Technologies) and used for subsequent PCR reaction. Platinum TAQ (Life Technologies) was used for semi-quantitative RT-PCR. Real time qPCR was performed using TaqMan universal master mix II (Life Technologies) and different TaqMan assays. A complete list of primers and assays for RT-PCR and qPCR is provided in S2 Table.

### Adenovirus, lentiviruses and cell transduction

Murine Xbp-1s and murine Atf4 adenoviruses used here were previously described[32, 33]. The shRNA lentivirus plasmids were packaged by transfection into 293T cells, as already described[34]. Stably transduced cells were selected in media containing puromycin (5μg/ml). Conditional shRNA were selected and maintained in media containing tetracycline-free serum (Clontech).

### Luciferase vectors and assays

The putative *NLRP1* promoter region 2555bp upstream of the ATG codon (chr17:5583958–5586512, assembly hg38), including 555 nucleotides corresponding to the 5′ untranslated region (5′UTR) was cloned from human genomic DNA (Promega) into the pGL4.12 luciferase vector (Promega) by PCR (PfuUltra#600380, Stratagene) using the KpnI and SacI restriction sites. Four shorter constructs were cloned using internal primers (S3 Table). All plasmid cloning was verified by DNA sequencing. Luciferase vectors, together with the renilla plasmid pGL4.74 (Promega) were transiently co-transfected (1:10 luciferase/renilla ratio) into HeLa cells using JetPrime (Polyplus transfection) according to the manufacturer’s protocol. After 24 hours, cells were treated with 2μM BFA overnight and luciferase signals were detected using the Dual-luciferase reporter assay system (Promega).

### Cell transfection, immunoprecipitation and immunoblot analysis

HeLa cells were seeded into 6-well plates at a density of 1 x 10^6^ cells per well. Two different siRNAs (25pmol each) were transfected using RNAiMAX reagent (Life Technologies) according to manufacturer’s protocol. After 24 hours, cells were treated overnight with 2μM BFA to induce ER stress. The following day the cells were washed twice with PBS and lysed in 1x SDS-sample buffer (62.5mM Tris-HCl pH6.8, 2% (w/v) SDS, 0.01% phenol red, 10% (w/v) glycerol and 20mM DTT). Endogenous NLRP1 protein was pulled-down in cells lysed with 50mM TRIS-HCl pH7.4, 150mM NaCl, 0.5% NP-40 and complete protease inhibitors (Roche). Clarified lysates were subjected to immunoprecipitation using protein G magnetic beads (DYNAL, Life Technologies) conjugated with NLRP1 antibody (R&D Systems). Lysates and pulled-down protein were subjected to SDS-PAGE/immunoblot analysis. Proteins were revealed using the LI-COR Odyssey system (LI-COR).

### Site-directed mutagenesis

Mutations in the *NLRP1* promoter-luciferase vector were generated by site-directed mutagenesis using the QuikChange® II XL kit from Agilent Technologies (Cat#200521). Primers were obtained using on line QuikChange® primer design tool. Primer sequences are listed in S3 Table. Plasmids were verified by DNA sequencing.

### Chromatin immunoprecipitation (ChIP)

HeLa cells (~1.2 x 10^6^) were treated overnight with DMSO or 2μM BFA. On the following day the cells were washed twice with PBS and cross-linked with 1% formaldehyde for 10 minutes at RT. Formaldehyde was quenched with 0.2M glycine for 10 minutes at RT. Cells were then lysed for 10 minutes with rotation at 4°C in 1ml buffer LB1 (50mM Hepes-KOH pH7.5, 140mM NaCl, 1mM EDTA, 10% glycerol, 0.5% NP-40 and 0.25% Triton X-100). Nuclei were pelleted and washed first with 1ml buffer LB2 (10mM Tris-HCl pH8.0, 200mM NaCl, 1mM EDTA pH8.0 and 0.5mM EGTA pH8.0) and then washed and resuspended in 750μl buffer LB3 (10mM Tris-HCl pH8.0, 200mM NaCl, 1mM EDTA pH8.0, 0.5mM EGTA pH8.0, 0.1% Na-deoxycholate, 0.5% N-lauroylsarcosine and protease inhibitors). Lysates were sonicated on ice with 10 seconds burst at 20% amplitude (Branson Sonifier S-450) with a 50 seconds pause between each cycle. Lysates were cleared by centrifugation and Triton X-100 was added to a final concentration of 1%. After removing 5% of the volume (input), the remainder was split into two aliquots and incubated overnight with 30μl protein G magnetic beads coated with antibodies. To coat the beads, they were equilibrated and washed in PBS with 0.5% BSA, incubated for 4 hours with ATF4 or control IgG antibody, and resuspended in buffer WB I (50mM Tris-HCl pH8.0, 150mM NaCl, 0.1% SDS, 0.1% Na-deoxycholate, 1% Triton X-100, 1mM EDTA). The beads were then washed once with WB I, once with WB II (50mM Tris-HCl pH8.0, 500mM NaCl, 0.1% SDS, 0.1% Na-deoxycholate, 1% Triton X-100, 1mM EDTA), once with WB III (10mM Tris-HCl pH8.0, 250mM LiCl, 0.5% NP-40, 0.5% Na-deoxycholate, 1mM EDTA) and twice with TE buffer. Beads were then eluted with 200μl 10mM Tris-HCl pH8.0, 0.5% SDS, 300mM NaCl and 5mM EDTA with shaking at 65°C for 30 minutes, and then were reverse cross-linked at 65°C overnight together with the input. RNA and proteins were removed by digestion with RNase at 37°C for 30 minutes and with proteinase K at 55°C for 1 hour. Finally, the DNA was purified using QIAquick columns (Qiagen) and resuspended in 40μl water. ChIP DNA was detected by qPCR using SYBR (Life Technologies) and specific primers for *NLRP1* and *ATF3* genomic promoter regions (S2 Table).

### RNA-sequencing

HeLa cells were treated with 5μg/ml tunicamycin for different time periods. Total RNA was extracted using an RNeasy kit (Qiagen) and reverse transcripts were generated using SuperScriptIII (Life Technologies) according to the manufacturer’s protocol. RNA-seq reads were first aligned to ribosomal RNA sequences to remove ribosomal reads. Remaining reads were aligned to the human reference genome (NCBI Build 37) using GSNAP, allowing maximum of 2 mismatches per 75 base sequence. Gene expression levels were quantified with RPKM values (reads mapping to a gene per kb of transcript per million reads sequenced) derived from the number of reads mapped to each RefSeq gene.

### CRISPR/Cas9 mediated genome editing

*ATF4* and *NLRP1* knock-out cells were generated using CRISPR/Cas9 technology. Guide RNAs (gRNA) with highest target specificity (S1 Table) were selected using the CRISPR design tool from MIT (http://crispr.mit.edu). Each gRNA was cloned in the pX330-U6-Chimeric_BB-CBh-hSpCas9 plasmid, a gift from Feng Zhang (Addgene plasmid #42230). Genome editing efficiency was evaluated using T7 Endonuclease I assay (NEB). *ATF4* ^*−/−*^ clones were generated by single cell sorting of EGFP-positive HeLa cells co-transfected with pX330-ATF4-gRNA and a EGFP plasmid (1:50 ratio) in 96-wells plates. After about two weeks, clones were treated with ER stress to induce ATF4 expression and screened by SDS-PAGE/immunoblot analysis (S7 Fig). To detect the presence of out-of-frame insertions/deletions (indels) in all *ATF4* alleles, the genotype of knock-out clones was verified by Sanger DNA sequencing.

To delete the entire 70 kb corresponding to *NLRP1* gene locus, HeLa, THP-1 and K562 cells were simultaneously co-transfected with 2 different CRISPR-Cas9 plasmids targeting the regions in proximity of the start codon (ATG) and of the stop codon (TGA) respectively, together with an EGFP plasmid to allow single cells sorting. After 2 weeks, clones were screened by genomic PCR. All *NLRP1* ^*−/−*^ clones were identified by the presence of PCR amplification using a forward primer in the 5’UTR and a reverse primer in the 3’UTR and by the contemporary absence of amplification products in each of the 5’UTR and the 3’UTR regions (S3 Fig). Locus deletion was verified by Sanger DNA sequencing and mRNA expression was analyzed by RT-PCR/qPCR. All gene knock-out clones are available upon request.

## Results

### NLRP1 is up-regulated during ER stress conditions

**Fig 1:**
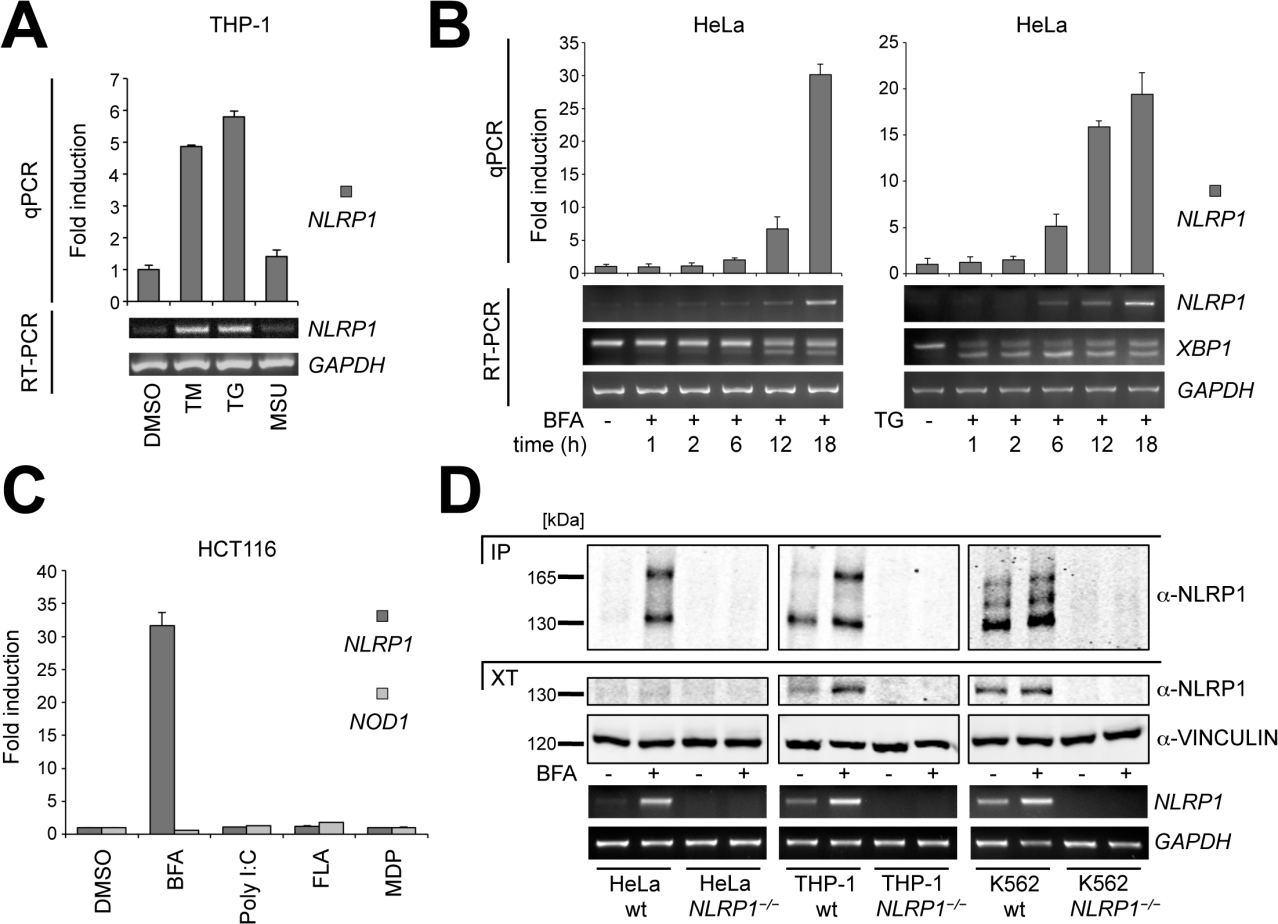
NLRP1 mRNA and protein are up-regulated upon ER stress. (A) Un-differentiated THP-1 cells were treated with the indicated stimuli for 6 hours. NLRP1 levels were measured by quantitative real-time PCR (qPCR) using cyclophillin A as an endogenous control. Semi-quantitative RT-PCR using a different NLRP1 primer set and GAPDH as a control is also shown. (B) HeLa cells were treated either with BFA or TG for the indicated times. NLRP1 mRNA levels were measured by qPCR and RT-PCR. Spliced and un-spliced XBP-1 forms were also evaluated by RT-PCR. (C) HCT116 cells were treated with the indicated stimuli for 24 hours. NLRP1 and NOD1 mRNA levels were measured by qPCR. (D) Cell lysates from wild-type or *NLRP1* ^*−/−*^ HeLa, THP-1 and K562 cells, untreated or treated with BFA for 20 hours, were normalized for total protein content. Cell extracts were then subjected to SDS-PAGE/immunoblot analysis before and after immunoprecipitation with NLRP1 antibody. Vinculin was detected as loading control. NLRP1 mRNA levels were also measured by RT-PCR. Each panel is representative of at least three independent experiments. (DMSO: dimethyl sulfoxide, TM: tunicamycin, TG: thapsigargin, MSU: monosodium urate crystals, BFA: brefeldin A, PolyI:C: polyinosinic-polycytidylic acid, FLA: flagellin, MDP: muramyl dipeptide, R837: Imiquimod)

Since ER stress was shown previously to activate the NLRP3 inflammasome[26, 27, 35], we explored the effects of ER stress on *NLRP1* gene expression. Stimulation of the human monocytic cell line THP-1 with two different ER stress inducers, tunicamycin (TM)—to inhibit N-linked glycosylation—and thapsigargin (TG)—to inhibit the ATP-dependent calcium pump SERCA-, produced marked increases in NLRP1 mRNA expression (Fig 1A). In contrast, the well-known NLRP3 inflammasome activator uric acid (MSU)[30] did not. We further tested ER stress-dependent NLRP1 induction in Jurkat cells, a human T cell line that normally does not express NLRP3 but has elevated NLRP1 mRNA basal levels (S1A Fig). The ER-Golgi transport blocker brefeldin A (BFA) and TG both induced time-dependent increases in NLRP1 mRNA levels in Jurkat cells (S1B Fig), while the TLR7 ligand and NLRP3 inflammasome activator R837[36] had no effect on NLRP1 mRNA expression. Similarly, time-dependent NLRP1 up-regulation was observed in HeLa epithelial cancer cells that have very low NLRP1 mRNA basal levels (Fig 1B). Time-course experiments with either BFA or TG showed that ER stress increased NLRP1 expression after 12 or 6 hours respectively (Fig 1B).

To test whether ER stress specifically induces *NLRP1* gene expression, we stimulated HCT116 human colon cancer cells with various inflammatory stimuli and measured mRNA expression of both NLRP1 and NOD1 (NLRC1), which is another human NLR family member. Induction of *NLRP1* but not *NOD1* gene expression was observed only during ER stress conditions induced by BFA treatment but not by other pro-inflammatory stimuli (Fig 1C). Next, to perform a more comprehensive analysis, we stimulated HeLa cells with TM for different times and performed a transcriptome study by total RNA-sequencing (RNA-seq). We focused on all currently known human NLR genes and found that among the 17 NLRs only NLRP1 and NLRC5 were expressed at any time point, while NLRP1 alone showed up-regulation under ER stress conditions (S2 Fig).

We next investigated whether increased NLRP1 mRNA levels upon ER stress correlate with increased NLRP1 protein expression. Immunoblot analysis of cell extracts from BFA-treated HeLa, THP-1 and K562 cells showed up-regulation of a 130 kDa band (Fig 1D), presumably corresponding to NLRP1 N-terminal autocleaved fragment[34]. Since a barely visible 130 kDa band was detected in HeLa cells after BFA treatment, we pulled-down endogenous NLRP1 protein. Immunoblot analysis revealed specific up-regulation of both cleaved and un-cleaved (around 165 kDa) fragments of NLRP1 during ER stress conditions in all cell lines, with the addition of an intermediate cleavage product in K562 cells (Fig 1D). To confirm antibody specificity, we utilized HeLa, THP-1 and K562 cells in which *NLRP1* gene locus was deleted using CRISPR-Cas9 technology (S3 Fig).

Taken together, these results show that perturbations of ER homeostasis in a variety of human cell lines specifically increase NLRP1 expression but not other NLR family genes.

### IRE1α and PERK, but not ATF6, induce NLRP1 expression

**Fig 2:**
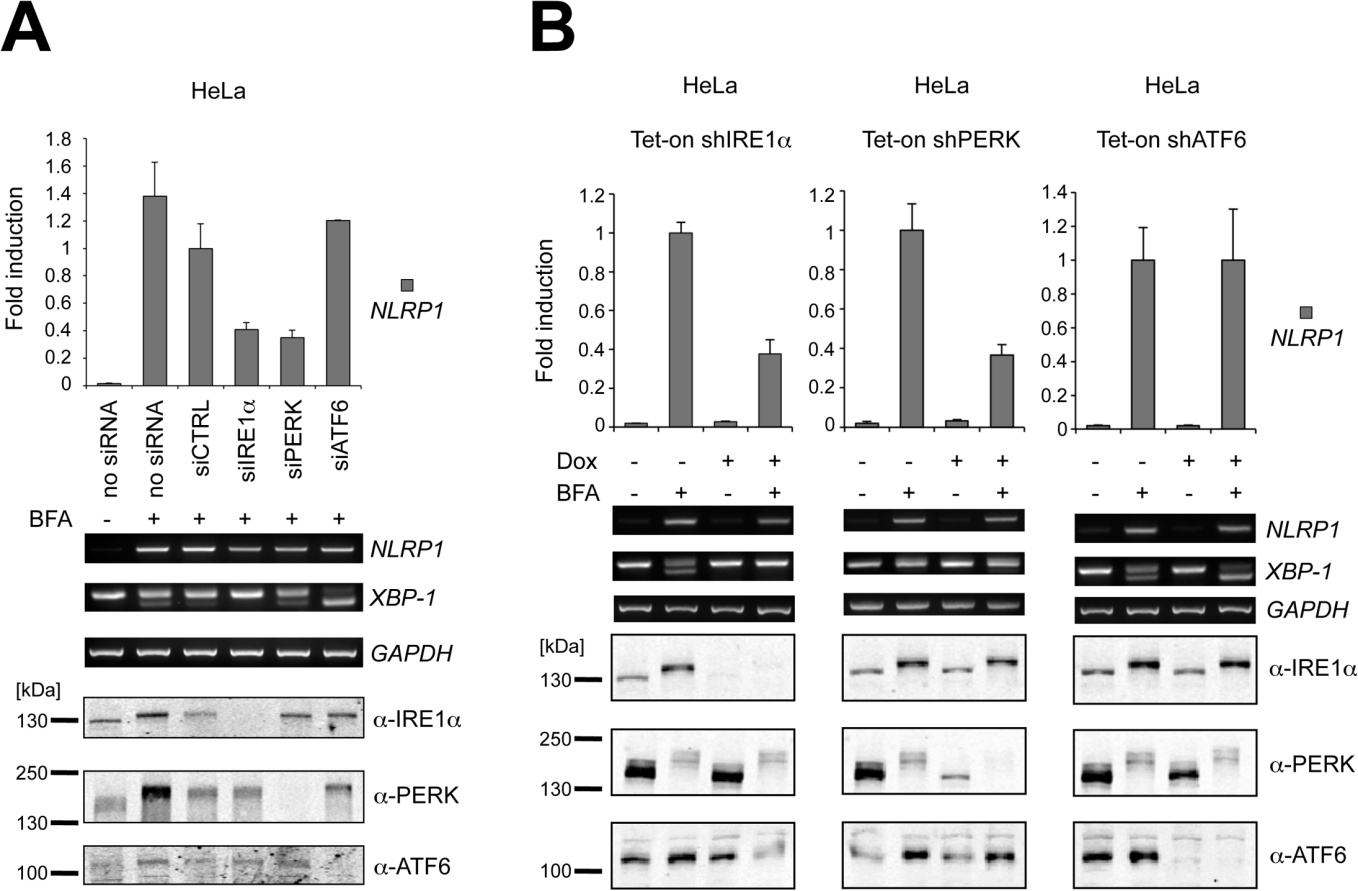
NLRP1 mRNA up-regulation is dependent on both IRE1α and PERK pathways. (A) IRE1α, PERK and ATF6 levels were reduced using siRNA. Upon treatment with ER stress, mRNA levels were measured by qPCR and RT-PCR. IRE1α, PERK and ATF6 knock-down was verified by SDS-PAGE/immunoblotting. (B) Stably transduced HeLa cells were cultured in presence or absence of doxycycline (Dox) for 24 hours and then treated overnight with 2μM BFA. mRNA levels were measured by qPCR and RT-PCR. IRE1α, PERK and ATF6 knock-down was verified by SDS-PAGE/immunoblotting. Each panel is representative of at least three independent experiments.

Three main pathways become activated during ER stress conditions, namely those involving IRE1α, PERK and ATF6. To verify whether these pathways could be involved in NLRP1 regulation, HeLa cells were transfected with a combination of two different siRNA for IRE1α, PERK and ATF6 and then stimulated overnight with the ER stress inducer, BFA. Experimentally reducing either IRE1α or PERK but not ATF6 levels resulted in diminished ER stress-induced *NLRP1* gene expression (Fig 2A).

To confirm these results, we derived HeLa cells wherein shRNA-mediated suppression of IRE1α, PERK or ATF6 could be conditionally induced using doxycycline (Fig 2B). Doxycycline-mediated knock-down of IRE1α and PERK but not ATF6 decreased NLRP1 mRNA induction upon BFA-induced ER stress, suggesting that a combination of transcription factors downstream of IRE1α (such as XBP-1s) and PERK (such as ATF4) activates the *NLRP1* promoter during ER stress conditions. The specificity of silencing each of the UPR pathways was verified by evaluating the expression of genes known to be regulated entirely by only one pathway[37], namely ERdj4 by IRE1α, ATF3 by PERK, and BIP by ATF6 (S4 Fig). Notably, ATF6 down-regulation resulted in an increased activation of IRE1α endoribonuclease activity as demonstrated by the increased XBP-1 splicing (Fig 2A and 2B) and ERdj4 expression (S4 Fig), which is in agreement with previous studies[38], but interestingly did not affect NLRP1 mRNA levels. These results indicate that both IRE1α and PERK but not ATF6 contribute to *NLRP1* gene induction during ER stress. However, the observations regarding the indirect effects of ATF6 on XBP-1s imply that XBP-1s is not the downstream mediator of IRE1α’s contributions to *NLRP1* gene expression.

### ATF4 but not XBP-1s stimulates NLRP1 up-regulation during ER stress

Because ATF4 mRNA is latent in cells, becoming actively translated only after induction of ER stress conditions, we tested whether protein biosynthesis is required for NLRP1 induction. Using cycloheximide (CHX), we found that new protein synthesis is necessary for up-regulating *NLRP1* gene expression after ER stress induction (S5A Fig). Interestingly, unconventional splicing of XBP-1 mRNA also required protein synthesis (S5A Fig), thus these CHX experiments did not differentiate between the IRE1α/XBP-1s versus PERK/ATF4 pathways. Furthermore, two different transcriptional inhibitors, actinomycin D (ActD) and 5,6-dichlorobenzimidazole 1-beta-D-ribofuranoside (DRB), markedly reduced NLRP1 induction during BFA stimulation, suggesting that NLRP1 up-regulation caused by ER stress is due to transcriptional control and not to increased mRNA stability (S5B Fig).

**Fig 3:**
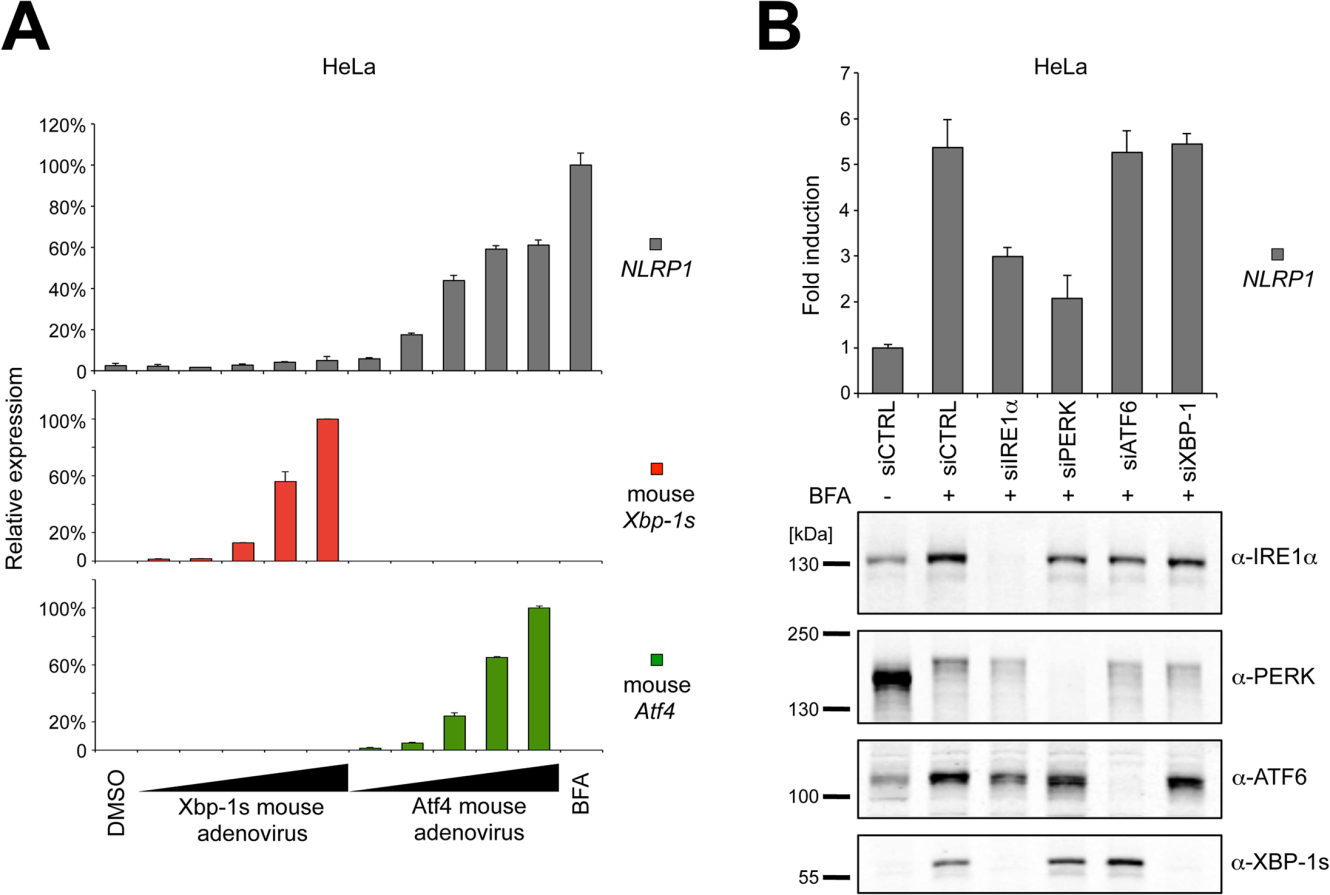
Atf4 but not Xbp-1s stimulates *NLRP1* gene expression during ER stress. (A) HeLa cells were infected with increasing concentrations of murine Xbp-1s and Atf4 adenovirus for 24 hours and NLRP1 mRNA was measured by qPCR. (B) IRE1α, PERK, ATF6 and XBP-1s were down-regulated using siRNA in HeLa cells. Cells were treated with BFA for 20 hours and mRNA levels were measured by qPCR. IRE1α, PERK, ATF6 and XBP-1s knock-down was verified by SDS-PAGE/immunoblotting. Each panel is representative of at least three independent experiments.

Because XBP-1s and ATF4 are transcription factors generated downstream of IRE1α and PERK, we experimentally determined whether either ATF4 or XBP-1s overexpression is sufficient to induce NLRP1 mRNA transcription. Using murine versions of cDNAs encoding these transcription factors to discriminate them from the endogenous human proteins, we infected HeLa cells with increasing doses of Atf4 or Xbp-1s adenovirus for 24 hours and then analyzed *NLRP1* gene expression by qPCR. We found that murine Atf4 overexpression was sufficient to stimulate NLRP1 mRNA increases in a dose-dependent manner (Fig 3A). In contrast, murine Xbp-1s overexpression—although able to induce human *ERdj4* gene expression (S6 Fig)—did not affect NLRP1 mRNA levels, suggesting that IRE1α’s contributions to NLRP1 expression may involve other downstream components of IRE1α signaling other than XBP-1s. This possibility was also supported by the lower levels of NLRP1 mRNA up-regulation that are mediated by Atf4 adenovirus compared to BFA induction (Fig 3A), suggesting that ATF4 may be acting in concert with other transcription factors. It is also possible that phosphorylation, acetylation or other post-translational modifications of ATF4 or other transcription factors are required for their full effects on *NLRP1* gene expression.

Although Xbp-1s alone was insufficient to induce NLRP1 expression in HeLa cells, we wondered whether it was necessary for NLRP1 induction by depleting XBP-1s using siRNA (Fig 3B). Down-regulation of XBP-1s expression, in contrast to IRE1α reduction, did not affect NLRP1 induction mediated by BFA, confirming that other factors controlled by IRE1α activation are required for stimulating *NLRP1* gene expression (Fig 3B).

Collectively, these results showed that XBP-1s is neither sufficient nor necessary for *NLRP1* gene induction. As such, some other IRE1α downstream effector appears to be responsible for promoting NLRP1 up-regulation in concert with the ATF4 transcription factor during ER stress.

### ATF4 transcription factor binds and activates the NLRP1 promoter during ER stress conditions

**Fig 4:**
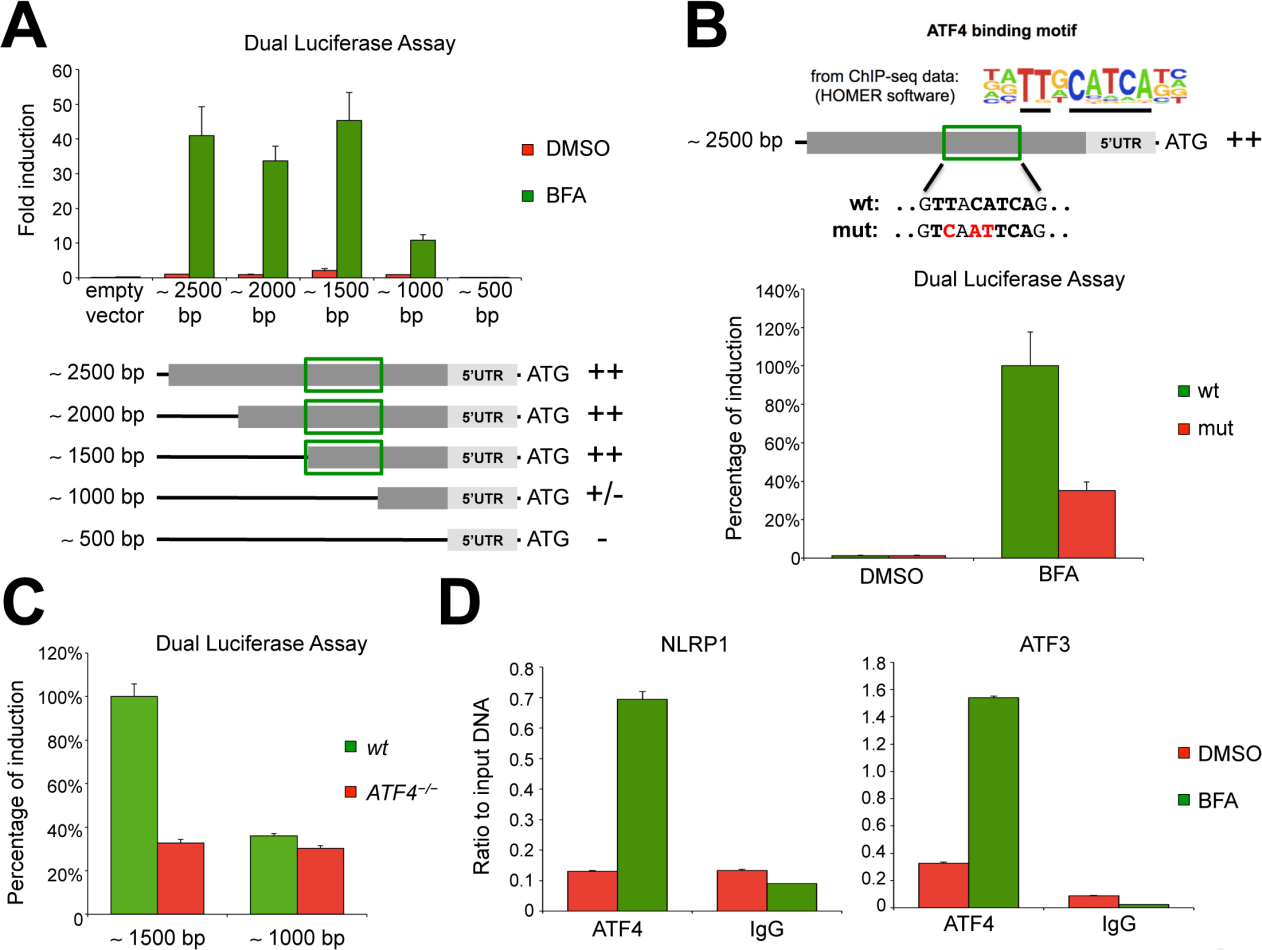
The transcription factor ATF4 binds and regulates the *NLRP1* gene promoter. (A) Different *NLRP1* promoter regions were cloned into a luciferase reporter vector. At 24 hours after transfection, HeLa cells were treated with BFA for 20 hours before measuring luciferase activity. (B) HeLa cells were transfected with wild-type *NLRP1* promoter or a version carrying mutations in the ATF4-binding motif. At 24 hours after transfection, cells were treated with BFA for 20 hours before measuring luciferase activity. (C) The indicated *NLRP1* promoter-luciferase vectors were transfected into either wild-type or *ATF4* ^*−/−*^ HeLa cells. At 24 hours after transfection, cells were treated with BFA for 20 hours before measuring luciferase activity. (D) ChIP analysis of un-stimulated (DMSO) or BFA-stimulated HeLa cells, followed by qPCR analysis of ATF4 occupancy at the NLRP1 and ATF3 promoter. ATF3 was used as positive control. Each panel is representative of at least three independent experiments.

We next focused on the genomic locus corresponding to the promoter of the human *NLRP1* gene and inserted this region into a plasmid containing a luciferase reporter gene. We also generated four different shorter *NLRP1* promoter constructs (Fig 4A) to localize the crucial region that could be responsible for ER stress responsiveness. Negligible luciferase signals were observed upon transfection of the NLRP1-reporter vectors into unstimulated HeLa cells, consistent with the very low basal expression level of NLRP1 in these cells (S1A Fig). In contrast, a remarkable induction was measured after ER stress caused by BFA (Fig 4A). Similar differences between untreated versus BFA treated samples were detected using shorter promoter constructs, in particular with the reporter vectors carrying 2000 and 1500bp segments from the human *NLRP1* promoter. Notably, upon transfection with a 1000bp segment of the *NLRP1* promoter, we observed > 70% reduction of the luciferase signal compared to plasmids expressing longer promoter sequences, suggesting that the *NLRP1* promoter region between 1500bp and 1000bp upstream of the ATG (green rectangle, Fig 4A) contains a critical ER stress-dependent responsive element. In contrast, while a luciferase reporter construct containing 1000bp upstream of the translation start site retained some ER stress induction (~30% relative to 2000bp construct), no ER stress-inducible luciferase activity was detected using a 500bp luciferase vector, which corresponds to the 5′UTR region (Fig 4A). Taken together, these data suggest the presence of a major and a minor ER stress responsive element located between 1500 and 1000bp and between 1000 and 500bp, respectively, upstream of the ATG translation start site in the *NLRP1* gene (Fig 4A).

Since ATF4 overexpression was sufficient to induce *NLRP1* gene expression, we sought to determine whether ATF4 directly regulates NLRP1 expression. Han *et al*. recently used chromatin-immunoprecipitation followed by next generation sequencing (ChIP-seq) to perform an exhaustive analysis of mouse genes that are regulated by Atf4 during ER stress[32]. Although this study did not identify any significant Atf4 sequencing peak in the promoter regions of mouse *Nlrp1a*, *Nlrp1b* or *Nlrp1c*, we decided to re-analyze their ChIP-seq raw data with the publicly available next-generation sequencing analysis software HOMER[39] and confirmed the Atf4 binding motif identified by the nucleotide sequence: TT-A/G-CATCA (Fig 4B). Thus, we searched the human *NLRP1* promoter region for the presence of an Atf4 consensus site. Interestingly, we found only one match in the middle of the region that we identified as containing a putative ER stress-responsive element (Fig 4A and 4B). We therefore used a mutagenesis approach to modify three nucleotides of the Atf4 binding motif in the luciferase vector carrying a 2500bp segment from the *NLRP1* promoter. When HeLa cells were transfected with the wild-type or ATF4 consensus mutant luciferase vectors, a significant reduction in the luciferase signal was observed in cells transfected with the mutated *NLRP1* promoter as compared to cells carrying the wild-type promoter following ER stress induction by BFA (Fig 4B).

To confirm these data, we generated a HeLa *ATF4* knock-out cell line using CRISPR-Cas9 technology (S7 Fig). ER stress-mediated luciferase induction of the 1500bp *NLRP1* promoter region was significantly reduced in *ATF4* ^*−/−*^ HeLa cells compared to wild-type cells (Fig 4C). In contrast, the residual luciferase activity induced by ER stress using the 1000bp of *NLRP1* promoter was unaffected by loss of ATF4 (Fig 4C). These data thus identify ATF4 transcription factor as an activator of *NLRP1* gene during ER stress conditions.

To extend the analysis of ATF4 effects on *NLRP1* expression, we performed ChIP experiments to study stimulation-dependent binding of the ATF4 transcription factor to the human *NLRP1* promoter. Consistent with our previous experiments, we observed substantial recruitment of ATF4 to the *NLRP1* promoter only upon BFA treatment, while no *NLRP1* DNA enrichment was detected in the negative IgG control (Fig 4D—left panel). Under the same ER stress conditions, we observed ATF4 binding to the *ATF3* promoter region, which was used as a positive control (Fig 4D—right panel).

In conclusion, our results demonstrate that upon ER stress the ATF4 transcription factor binds and activates the human *NLRP1* promoter. Other transcription factors or other signaling mechanisms, particularly those acting downstream of IRE1α, may also contribute to *NLRP1* gene expression during ER stress and they are currently under investigation.

## Discussion

Multiple members of the NLR family are capable of forming so-called “inflammasomes”, which are multi-protein complexes that recruit and activate pro-inflammatory caspases and stimulate proteolytic activation of cytokines such as Interleukin-1β (IL-1β) and Interleukin-18 (IL-18). Prior studies have recently demonstrated NLRP3 inflammasome activation in response to ER stress conditions[26, 27, 35]. In this study, we present the first evidence that expression of the human *NLRP1* gene is transcriptionally up-regulated in response to perturbations of the ER. The human NLRP1 protein is also capable of forming inflammasomes that stimulate caspase-1 activation and processing of pro-IL-1β[29]. Interestingly, hereditary polymorphisms in the human *NLRP1* gene have been associated with vitiligo, autoimmunity, systemic sclerosis, and increases sensitivity of leprosy[40–42].

We showed that both the IRE1α and PERK pathways are important for ER stress-induced *NLRP1* gene expression. Indeed, we demonstrated that ATF4 transcription factor (which is produced downstream of PERK) binds and stimulates the human *NLRP1* promoter upon ER stress induction. Other transcription factors downstream of the IRE1α-TRAF2 pathway, such as *RELB* and *NFKB1*, may also be involved in this process and they are currently under investigation. In contrast, XBP-1s (the best-studied transcription factor produced by IRE1α-mediated mRNA splicing during the initial restorative phase of the UPR) did not participate in NLRP1 regulation, even though it has been described to play a role in inflammatory responses[18]. This finding, together with the observation that NLRP1 expression is induced at later times compared to XBP-1 splicing (Fig 1B), suggest that *NLRP1* gene expression is stimulated during the terminal phase of the UPR.

Interestingly, we observed NLRP1 up-regulation in many different cell lines in which one or more components of the inflammasomes, such as NLRP3 or the adaptor protein ASC, are not expressed (S1A Fig) and are not induced during ER stress (data not shown). Together with the observation that most of these cell lines also do not express or up-regulate following ER stress the pro-forms of IL-1β or IL-18, this finding strongly suggests that NLRP1 function during ER stress is not related to pro-inflammatory cytokine secretion but presumably to other functions such as caspases activation. In this regard, NLRP1 was initially described to be involved in apoptosis[43]. In addition, the NLRP1-interacting human proteins caspase-4 and caspase-5[29] have been shown to become activated during ER stress[44, 45] and were more recently implicated in cell death induced in response to intracellular bacterial components[46]. Whether the human NLRP1 inflammasome is also involved in ER stress-dependent caspase-4/5 activation and cell death in response to bacterial infections is currently under investigation. Additionally, ischemia-reperfusion injury in the brain (which is a known cause of ER stress) has been shown to cause increases in NLRP1 expression under conditions where caspase-1 inhibitors show neuroprotective activity[47].

ATF4 is a member of the ATF/CREB (activating transcription factor/cyclic AMP response element binding protein) family of basic region-leucine zipper (bZip) transcription factors[48]. Notably, the transcription factor CREB has been described to induce NLRP1 expression in leukemia cells[49]. However, we did not observe any NLRP1 mRNA induction in HeLa cells upon treatment with the CREB activator forskolin (S8A Fig). In addition, specific siRNA against *CREB1* gene did not affect NLRP1 mRNA induction by BFA (S8B Fig), suggesting that CREB does not play a role in human *NLRP1* gene induction during ER stress conditions.

Genomic variations in the NLR family genes are extensive among mammalian species, suggesting that specialization of these innate immunity genes has occurred during evolution to adapt to differences in the pathogens that various species encounter in their environmental niches. In this regard, the closest analog of the human *NLRP1* gene in mice is a cluster of genes that vary among mouse strains in the proteins they encode and that lack the N-terminal PYRIN domain found in the human NLRP1 protein[50]. Whether the murine gene locus showing homology to human *NLRP1* is also subject to regulation by ER stress remains to be determined, however, a murine genome-wide ChIP-seq analysis failed to reveal ATF4 binding sites[32].

In conclusion, this study demonstrates that the human *NLRP1* gene is up-regulated during severe ER stress conditions. Furthermore, we clarified a portion of the underlying mechanism, which involves direct binding of transcription factor ATF4 to the *NLRP1* gene promoter. Future efforts will be directed to identifying the mechanisms by which the IRE1α pathway also makes contributions to ER stress-induced *NLRP1* gene expression and to understanding the roles played by the NLRP1 protein in the context of ER stress conditions.

## Supporting Information

S1 Fig Expression of inflammasome genes in cancer and leukemia cell lines subjected to ER stress. (A) Various mRNA levels were evaluated by RT-PCR. NLRP1 mRNA expression does not overlap with other inflammasome components such as NLRP3 or ASC. CARD8 mRNA was ubiquitously present in all cell lines tested. (B) Jurkat cells were treated with the indicated stimuli for various times, then mRNA levels were measured as in Fig 1A.
(TIFF)

S2 Fig Global expression analysis of NLRs during prolonged ER stress conditions. Transcript expression of NLRs genes was determined by RNA-seq from HeLa cells treated with 5μg/ml tunicamycin for various times. RNA-seq was performed in triplicates for each time point.
(TIFF)

S3 Fig *NLRP1* knock-out cell lines generated using CRISPR-Cas9. Schematic representation of *NLRP1* locus deletion. Cells were simultaneously co-transfected with 2 different CRISPR-Cas9 plasmids targeting near the 5’UTR and the 3’UTR respectively. By genomic DNA sequence analysis of the 5’UTR-3’UTR PCR product, all *NLRP1* ^*−/−*^ clones were determined to be homozygous in which all mutated alleles were re-ligated exactly in correspondence with the 2 predicted double-strand breaks. Allelic *NLRP1* deletions were identified by PCR amplification using a forward primer in the 5’UTR and a reverse primer in the 3’UTR. In this example, 3 full *NLRP1* ^*−/−*^ clones (red rectangles) were identified by the absence of both PCR amplification products in the 5’UTR and in the 3’UTR.
(TIFF)

S4 Fig ATF6 down-regulation increases XBP-1s-dependent ERdj4 expression. The specificity of silencing of IRE1α, PERK and ATF6 pathways was confirmed by monitoring respectively ERdj4, ATF3 and BIP gene expression by qPCR.
(TIFF)

S5 Fig NLRP1 induction requires new protein synthesis and mRNA transcription. (A) HeLa cells were pre-incubated with CHX for one hour before overnight induction of ER stress with BFA. Cells were collected the following day and NLRP1, XBP-1 and GAPDH levels were measured by RT-PCR. (B) HeLa cells were incubated with DMSO or BFA for a total of 8 hours. Additionally, cells were exposed to transcriptional inhibitors ActD or DRB for the final 3 or 6 hours of BFA treatment. NLRP1 mRNA levels were evaluated by qPCR.
(TIFF)

S6 Fig Overexpression of murine Xbp-1s and Atf4 induce ERdj4 and ATF3, respectively, in HeLa cells. Functional validation of murine Xbp-1s and Atf4 adenovirus was performed by measuring gene expression of downstream targets by qPCR. Cells were treated as in Fig 3A.
(TIFF)

S7 Fig *ATF4* knock-out HeLa cells generated using CRISPR-Cas9. Cell lysates from wild-type or *ATF4* ^*−/−*^ HeLa cells, untreated or treated with BFA for various times, were normalized for total protein content. Cell extracts were then subjected to SDS-PAGE/immunoblot analysis. Tubulin was detected as loading control.
(TIFF)

S8 Fig CREB1 is not involved in NLRP1 mRNA induction during ER stress. (A) HeLa cells were treated with either TG or Forskolin for the indicated times. NLRP1 and XBP-1 mRNA levels were evaluated by RT-PCR. (B) HCT116 cells were transfected with siRNA against CREB1 gene for 24 hours. Scrambled siRNA were used as control. Upon treatment with BFA for 16 hours, NLRP1 and XBP-1 mRNA levels were evaluated by RT-PCR.
(TIFF)

S1 Table List of siRNA, shRNA and CRISPR targets. (XLSX)

S2 Table List of primers and assays for RT-PCR and qPCR. (XLSX)

S3 Table List of primers for cloning and mutagenesis. (XLSX)

## References

[1] Braakman I, Bulleid NJ. Protein folding and modification in the mammalian endoplasmic reticulum. Annu Rev Biochem. 2011;80:71–99. Epub 2011/04/19. 10.1146/annurev-biochem-062209-093836 . 21495850

[2] Kaufman RJ, Malhotra JD. Calcium trafficking integrates endoplasmic reticulum function with mitochondrial bioenergetics. Biochim Biophys Acta. 2014;1843(10):2233–9. Epub 2014/04/03. doi: S0167-4889(14)00106-2 [pii] 10.1016/j.bbamcr.2014.03.022 . 24690484 PMC4285153

[3] Walter P, Ron D. The unfolded protein response: from stress pathway to homeostatic regulation. Science. 2011;334(6059):1081–6. Epub 2011/11/26. doi: 334/6059/1081 [pii] 10.1126/science.1209038 . 22116877

[4] Ozcan L, Tabas I. Role of endoplasmic reticulum stress in metabolic disease and other disorders. Annu Rev Med. 2012;63:317–28. Epub 2012/01/18. 10.1146/annurev-med-043010-144749 . 22248326 PMC3290993

[5] Ron D, Walter P. Signal integration in the endoplasmic reticulum unfolded protein response. Nat Rev Mol Cell Biol. 2007;8(7):519–29. Epub 2007/06/15. doi: nrm2199 [pii] 10.1038/nrm2199 . 17565364

[6] Kaufman RJ. Stress signaling from the lumen of the endoplasmic reticulum: coordination of gene transcriptional and translational controls. Genes Dev. 1999;13(10):1211–33. Epub 1999/05/27. . 10346810 10.1101/gad.13.10.1211

[7] Wang S, Kaufman RJ. The impact of the unfolded protein response on human disease. J Cell Biol. 2012;197(7):857–67. Epub 2012/06/27. doi: jcb.201110131 [pii] 10.1083/jcb.201110131 . 22733998 PMC3384412

[8] Hetz C, Martinon F, Rodriguez D, Glimcher LH. The unfolded protein response: integrating stress signals through the stress sensor IRE1alpha. Physiol Rev. 2011;91(4):1219–43. Epub 2011/10/21. 91/4/1219 [pii] 10.1152/physrev.00001.2011 . 22013210

[9] Tabas I, Ron D. Integrating the mechanisms of apoptosis induced by endoplasmic reticulum stress. Nat Cell Biol. 2011;13(3):184–90. Epub 2011/03/03. doi: ncb0311-184 [pii] 10.1038/ncb0311-184 . 21364565 PMC3107571

[10] Kim I, Xu W, Reed JC. Cell death and endoplasmic reticulum stress: disease relevance and therapeutic opportunities. Nat Rev Drug Discov. 2008;7(12):1013–30. Epub 2008/12/02. doi: nrd2755 [pii] 10.1038/nrd2755 . 19043451

[11] Hotamisligil GS. Endoplasmic reticulum stress and the inflammatory basis of metabolic disease. Cell. 2010;140(6):900–17. Epub 2010/03/23. doi: S0092-8674(10)00187-X [pii] 10.1016/j.cell.2010.02.034 . 20303879 PMC2887297

[12] Lindholm D, Wootz H, Korhonen L. ER stress and neurodegenerative diseases. Cell Death Differ. 2006;13(3):385–92. Epub 2006/01/07. 10.1038/sj.cdd.4401778 . 16397584

[13] He B. Viruses, endoplasmic reticulum stress, and interferon responses. Cell Death Differ. 2006;13(3):393–403. Epub 2006/01/07. doi: 4401833 [pii] 10.1038/sj.cdd.4401833 . 16397582

[14] Cho JA, Lee AH, Platzer B, Cross BC, Gardner BM, De Luca H, et al The unfolded protein response element IRE1alpha senses bacterial proteins invading the ER to activate RIG-I and innate immune signaling. Cell Host Microbe. 2013;13(5):558–69. Epub 2013/05/21. doi: S1931-3128(13)00154-6 [pii] 10.1016/j.chom.2013.03.011 . 23684307 PMC3766372

[15] Roy CR, Salcedo SP, Gorvel JP. Pathogen-endoplasmic-reticulum interactions: in through the out door. Nat Rev Immunol. 2006;6(2):136–47. Epub 2006/02/24. doi: nri1775 [pii] 10.1038/nri1775 . 16491138 PMC7097709

[16] Zhang K, Kaufman RJ. From endoplasmic-reticulum stress to the inflammatory response. Nature. 2008;454(7203):455–62. Epub 2008/07/25. doi: nature07203 [pii] 10.1038/nature07203 . 18650916 PMC2727659

[17] Kaser A, Lee AH, Franke A, Glickman JN, Zeissig S, Tilg H, et al XBP1 links ER stress to intestinal inflammation and confers genetic risk for human inflammatory bowel disease. Cell. 2008;134(5):743–56. Epub 2008/09/09. doi: S0092-8674(08)00941-0 [pii] 10.1016/j.cell.2008.07.021 . 18775308 PMC2586148

[18] Martinon F, Chen X, Lee AH, Glimcher LH. TLR activation of the transcription factor XBP1 regulates innate immune responses in macrophages. Nat Immunol. 2010;11(5):411–8. Epub 2010/03/31. doi: ni.1857 [pii] 10.1038/ni.1857 . 20351694 PMC3113706

[19] Qiu Q, Zheng Z, Chang L, Zhao YS, Tan C, Dandekar A, et al Toll-like receptor-mediated IRE1alpha activation as a therapeutic target for inflammatory arthritis. EMBO J. 2013;32(18):2477–90. Epub 2013/08/15. doi: emboj2013183 [pii] 10.1038/emboj.2013.183 . 23942232 PMC3770952

[20] Urano F, Wang X, Bertolotti A, Zhang Y, Chung P, Harding HP, et al Coupling of stress in the ER to activation of JNK protein kinases by transmembrane protein kinase IRE1. Science. 2000;287(5453):664–6. Epub 2000/01/29. doi: 8218 [pii]. . 10650002 10.1126/science.287.5453.664

[21] Kitamura M. Biphasic, bidirectional regulation of NF-kappaB by endoplasmic reticulum stress. Antioxid Redox Signal. 2009;11(9):2353–64. Epub 2009/02/04. 10.1089/ARS.2008.2391 . 19187000

[22] Harding HP, Zhang Y, Zeng H, Novoa I, Lu PD, Calfon M, et al An integrated stress response regulates amino acid metabolism and resistance to oxidative stress. Mol Cell. 2003;11(3):619–33. Epub 2003/04/02. doi: S1097276503001059 [pii]. . 12667446 10.1016/s1097-2765(03)00105-9

[23] Claudio N, Dalet A, Gatti E, Pierre P. Mapping the crossroads of immune activation and cellular stress response pathways. EMBO J. 2013;32(9):1214–24. Epub 2013/04/16. doi: emboj201380 [pii] 10.1038/emboj.2013.80 . 23584529 PMC3642686

[24] Medinas DB, Hetz C. Proteostasis impairment: at the intersection between Alzheimer's disease and diabetes. Cell Metab. 2013;18(6):771–2. Epub 2013/12/10. doi: S1550-4131(13)00458-0 [pii] 10.1016/j.cmet.2013.11.009 . 24315366

[25] Wen H, Miao EA, Ting JP. Mechanisms of NOD-like receptor-associated inflammasome activation. Immunity. 2013;39(3):432–41. Epub 2013/09/24. doi: S1074-7613(13)00389-0 [pii] 10.1016/j.immuni.2013.08.037 . 24054327 PMC3835203

[26] Lerner AG, Upton JP, Praveen PV, Ghosh R, Nakagawa Y, Igbaria A, et al IRE1alpha induces thioredoxin-interacting protein to activate the NLRP3 inflammasome and promote programmed cell death under irremediable ER stress. Cell Metab. 2012;16(2):250–64. Epub 2012/08/14. doi: S1550-4131(12)00284-7 [pii] 10.1016/j.cmet.2012.07.007 . 22883233 PMC4014071

[27] Oslowski CM, Hara T, O'Sullivan-Murphy B, Kanekura K, Lu S, Hara M, et al Thioredoxin-interacting protein mediates ER stress-induced beta cell death through initiation of the inflammasome. Cell Metab. 2012;16(2):265–73. Epub 2012/08/14. doi: S1550-4131(12)00282-3 [pii] 10.1016/j.cmet.2012.07.005 . 22883234 PMC3418541

[28] Masters SL, Gerlic M, Metcalf D, Preston S, Pellegrini M, O'Donnell JA, et al NLRP1 inflammasome activation induces pyroptosis of hematopoietic progenitor cells. Immunity. 2012;37(6):1009–23. Epub 2012/12/12. 10.1016/j.immuni.2012.08.027 23219391 PMC4275304

[29] Martinon F, Burns K, Tschopp J. The inflammasome: a molecular platform triggering activation of inflammatory caspases and processing of proIL-beta. Mol Cell. 2002;10(2):417–26. Epub 2002/08/23. doi: S1097276502005993 [pii]. . 12191486 10.1016/s1097-2765(02)00599-3

[30] Martinon F, Petrilli V, Mayor A, Tardivel A, Tschopp J. Gout-associated uric acid crystals activate the NALP3 inflammasome. Nature. 2006;440(7081):237–41. Epub 2006/01/13. doi: nature04516 [pii] 10.1038/nature04516 . 16407889

[31] Wiederschain D, Wee S, Chen L, Loo A, Yang G, Huang A, et al Single-vector inducible lentiviral RNAi system for oncology target validation. Cell Cycle. 2009;8(3):498–504. Epub 2009/01/30. doi: 7701 [pii]. . 19177017 10.4161/cc.8.3.7701

[32] Han J, Back SH, Hur J, Lin YH, Gildersleeve R, Shan J, et al ER-stress-induced transcriptional regulation increases protein synthesis leading to cell death. Nat Cell Biol. 2013;15(5):481–90. Epub 2013/04/30. doi: ncb2738 [pii] 10.1038/ncb2738 . 23624402 PMC3692270

[33] Wang S, Chen Z, Lam V, Han J, Hassler J, Finck BN, et al IRE1alpha-XBP1s induces PDI expression to increase MTP activity for hepatic VLDL assembly and lipid homeostasis. Cell Metab. 2012;16(4):473–86. Epub 2012/10/09. doi: S1550-4131(12)00363-4 [pii] 10.1016/j.cmet.2012.09.003 . 23040069 PMC3569089

[34] D'Osualdo A, Weichenberger CX, Wagner RN, Godzik A, Wooley J, Reed JC. CARD8 and NLRP1 undergo autoproteolytic processing through a ZU5-like domain. PLoS One. 2011;6(11):e27396 Epub 2011/11/17. 10.1371/journal.pone.0027396 PONE-D-11-15100 [pii]. . 22087307 PMC3210808

[35] Menu P, Mayor A, Zhou R, Tardivel A, Ichijo H, Mori K, et al ER stress activates the NLRP3 inflammasome via an UPR-independent pathway. Cell Death Dis. 2012;3:e261. Epub 2012/01/27. doi: cddis2011132 [pii] 10.1038/cddis.2011.132 . 22278288 PMC3270266

[36] Kanneganti TD, Ozoren N, Body-Malapel M, Amer A, Park JH, Franchi L, et al Bacterial RNA and small antiviral compounds activate caspase-1 through cryopyrin/Nalp3. Nature. 2006;440(7081):233–6. Epub 2006/01/13. doi: nature04517 [pii] 10.1038/nature04517 . 16407888

[37] Shoulders MD, Ryno LM, Genereux JC, Moresco JJ, Tu PG, Wu C, et al Stress-independent activation of XBP1s and/or ATF6 reveals three functionally diverse ER proteostasis environments. Cell Rep. 2013;3(4):1279–92. Epub 2013/04/16. doi: S2211-1247(13)00131-9 [pii] 10.1016/j.celrep.2013.03.024 . 23583182 PMC3754422

[38] Wu J, Rutkowski DT, Dubois M, Swathirajan J, Saunders T, Wang J, et al ATF6alpha optimizes long-term endoplasmic reticulum function to protect cells from chronic stress. Dev Cell. 2007;13(3):351–64. Epub 2007/09/04. doi: S1534-5807(07)00266-3 [pii] 10.1016/j.devcel.2007.07.005 . 17765679

[39] Heinz S, Benner C, Spann N, Bertolino E, Lin YC, Laslo P, et al Simple combinations of lineage-determining transcription factors prime cis-regulatory elements required for macrophage and B cell identities. Mol Cell. 2010;38(4):576–89. Epub 2010/06/02. doi: S1097-2765(10)00366-7 [pii] 10.1016/j.molcel.2010.05.004 . 20513432 PMC2898526

[40] Jin Y, Mailloux CM, Gowan K, Riccardi SL, LaBerge G, Bennett DC, et al NALP1 in vitiligo-associated multiple autoimmune disease. N Engl J Med. 2007;356(12):1216–25. Epub 2007/03/23. doi: 356/12/1216 [pii] 10.1056/NEJMoa061592 . 17377159

[41] Dieude P, Guedj M, Wipff J, Ruiz B, Riemekasten G, Airo P, et al NLRP1 influences the systemic sclerosis phenotype: a new clue for the contribution of innate immunity in systemic sclerosis-related fibrosing alveolitis pathogenesis. Ann Rheum Dis. 2011;70(4):668–74. Epub 2010/12/15. doi: ard.2010.131243 [pii] 10.1136/ard.2010.131243 . 21149496

[42] Pontillo A, Laurentino W, Crovella S, Pereira AC. NLRP1 haplotypes associated with leprosy in Brazilian patients. Infect Genet Evol. 2013;19:274–9. Epub 2013/06/19. doi: S1567-1348(13)00231-1 [pii] 10.1016/j.meegid.2013.06.006 . 23770116

[43] Chu ZL, Pio F, Xie Z, Welsh K, Krajewska M, Krajewski S, et al A novel enhancer of the Apaf1 apoptosome involved in cytochrome c-dependent caspase activation and apoptosis. J Biol Chem. 2001;276(12):9239–45. Epub 2000/12/23. 10.1074/jbc.M006309200 M006309200 [pii]. . 11113115

[44] Hitomi J, Katayama T, Eguchi Y, Kudo T, Taniguchi M, Koyama Y, et al Involvement of caspase-4 in endoplasmic reticulum stress-induced apoptosis and Abeta-induced cell death. J Cell Biol. 2004;165(3):347–56. Epub 2004/05/05. 10.1083/jcb.200310015 jcb.200310015 [pii]. . 15123740 PMC2172196

[45] Bian ZM, Elner SG, Khanna H, Murga-Zamalloa CA, Patil S, Elner VM. Expression and functional roles of caspase-5 in inflammatory responses of human retinal pigment epithelial cells. Invest Ophthalmol Vis Sci. 2011;52(12):8646–56. Epub 2011/10/05. doi: iovs.11-7570 [pii] 10.1167/iovs.11-7570 . 21969293 PMC3230287

[46] Shi J, Zhao Y, Wang Y, Gao W, Ding J, Li P, et al Inflammatory caspases are innate immune receptors for intracellular LPS. Nature. 2014;514(7521):187–92. Epub 2014/08/15. doi: nature13683 [pii] 10.1038/nature13683 . 25119034

[47] Fann DY, Lee SY, Manzanero S, Tang SC, Gelderblom M, Chunduri P, et al Intravenous immunoglobulin suppresses NLRP1 and NLRP3 inflammasome-mediated neuronal death in ischemic stroke. Cell Death Dis. 2013;4:e790. Epub 2013/09/07. doi: cddis2013326 [pii] 10.1038/cddis.2013.326 . 24008734 PMC3789184

[48] Ameri K, Harris AL. Activating transcription factor 4. Int J Biochem Cell Biol. 2008;40(1):14–21. Epub 2007/05/01. doi: S1357-2725(07)00041-6 [pii] 10.1016/j.biocel.2007.01.020 . 17466566

[49] Sanz C, Calasanz MJ, Andreu E, Richard C, Prosper F, Fernandez-Luna JL. NALP1 is a transcriptional target for cAMP-response-element-binding protein (CREB) in myeloid leukaemia cells. Biochem J. 2004;384(Pt 2):281–6. Epub 2004/08/03. 10.1042/BJ20040867 BJ20040867 [pii]. . 15285719 PMC1134111

[50] Guey B, Bodnar M, Manie SN, Tardivel A, Petrilli V. Caspase-1 autoproteolysis is differentially required for NLRP1b and NLRP3 inflammasome function. Proc Natl Acad Sci U S A. 2014;111(48):17254–9. Epub 2014/11/19. doi: 1415756111 [pii] 10.1073/pnas.1415756111 . 25404286 PMC4260594

