# Supplementary figures and images for: Transcription Factor ATF4 Induces NLRP1 Inflammasome Expression during Endoplasmic Reticulum Stress
Source: PLoS One. 2015 Jun 18;10(6):e0130635. doi: 10.1371/journal.pone.0130635 (PMC4472728; doi:10.1371/journal.pone.0130635)

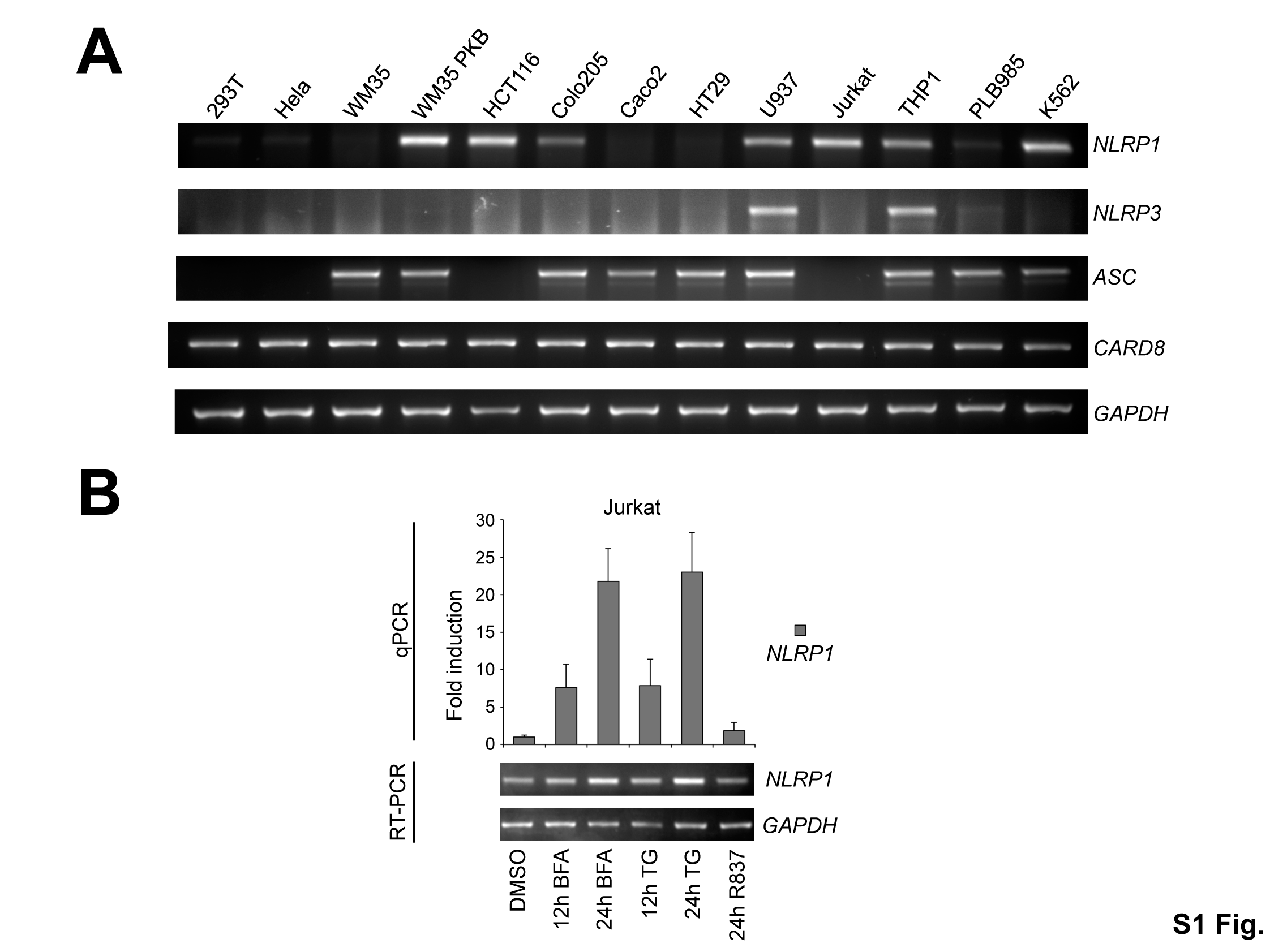

Supplement: S1 Fig — (A) Various mRNA levels were evaluated by RT-PCR. NLRP1 mRNA expression does not overlap with other inflammasome components such as NLRP3 or ASC. CARD8 mRNA was ubiquitously present in all cell lines tested. (B) Jurkat cells were treated with the indicated stimuli for various times, then mRNA levels were measured as in Fig 1A. (TIFF) [file pone.0130635.s001.tiff]

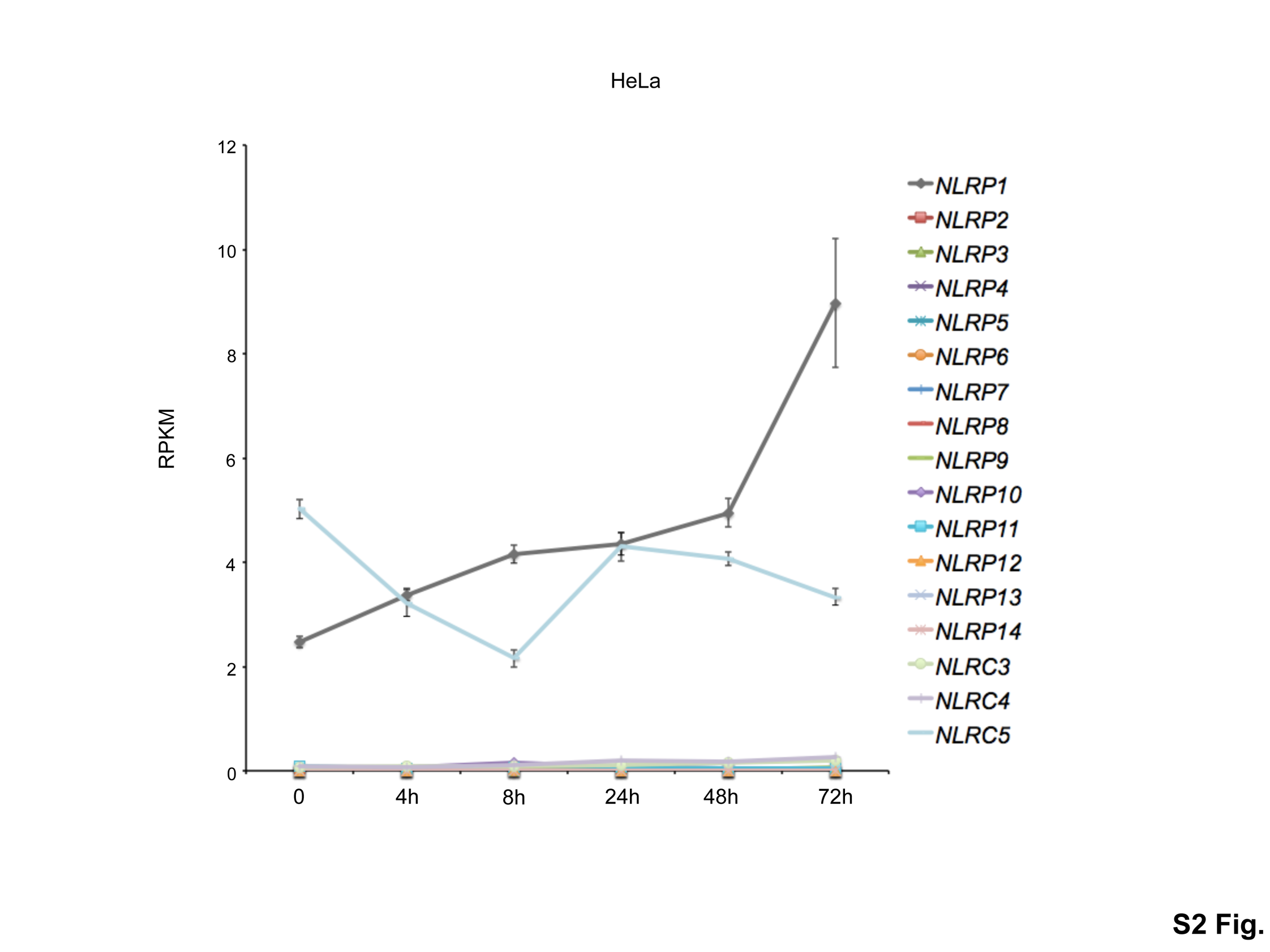

Supplement: S2 Fig — Transcript expression of NLRs genes was determined by RNA-seq from HeLa cells treated with 5μg/ml tunicamycin for various times. RNA-seq was performed in triplicates for each time point. (TIFF) [file pone.0130635.s002.tiff]

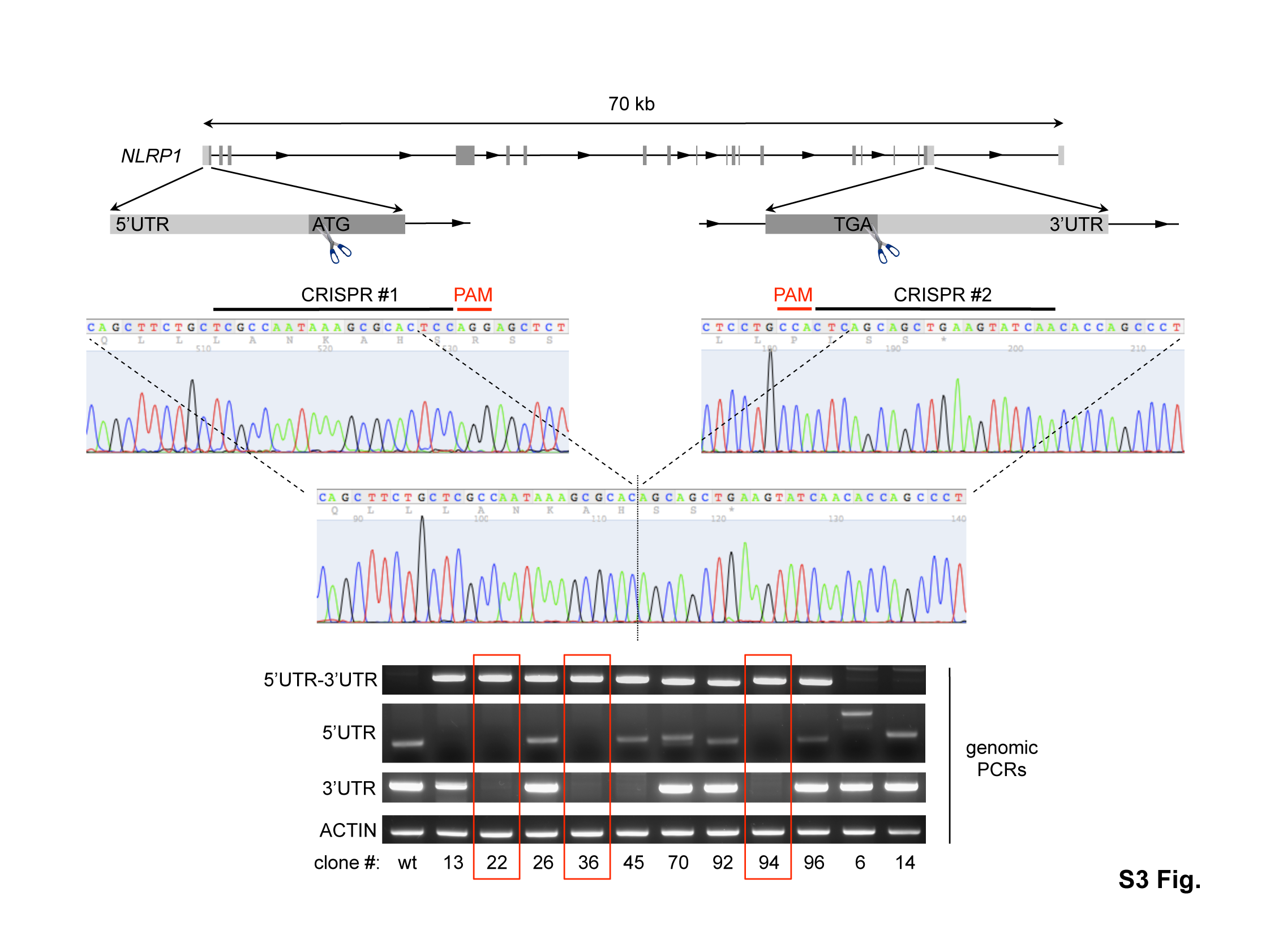

Supplement: S3 Fig — Schematic representation of NLRP1 locus deletion. Cells were simultaneously co-transfected with 2 different CRISPR-Cas9 plasmids targeting near the 5’UTR and the 3’UTR respectively. By genomic DNA sequence analysis of the 5’UTR-3’UTR PCR product, all NLRP1 −/− clones were determined to be homozygous in which all mutated alleles were re-ligated exactly in correspondence with the 2 predicted double-strand breaks. Allelic NLRP1 deletions were identified by PCR amplification using a forward primer in the 5’UTR and a reverse primer in the 3’UTR. In this example, 3 full NLRP1 −/− clones (red rectangles) were identified by the absence of both PCR amplification products in the 5’UTR and in the 3’UTR. (TIFF) [file pone.0130635.s003.tiff]

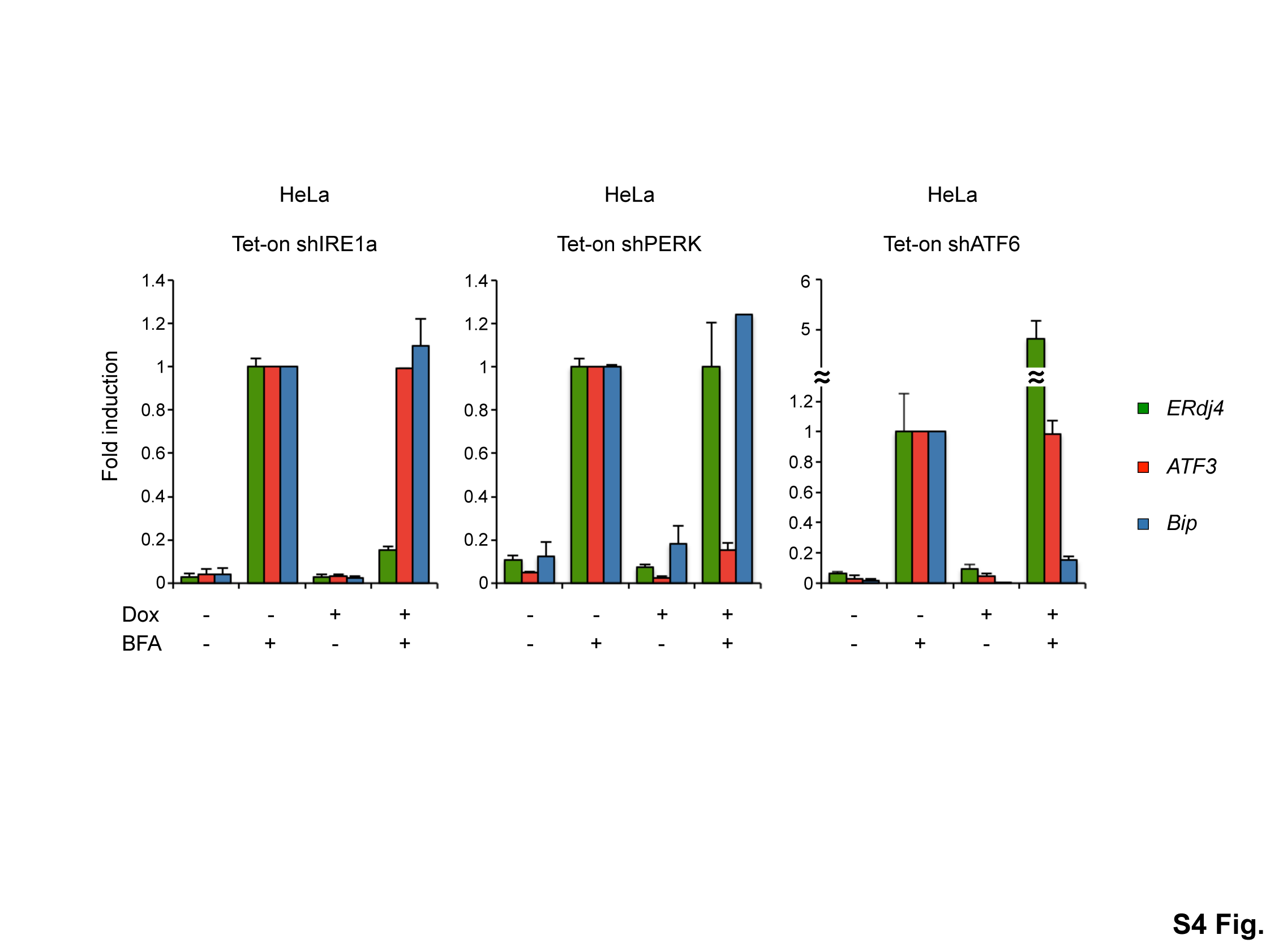

Supplement: S4 Fig — The specificity of silencing of IRE1α, PERK and ATF6 pathways was confirmed by monitoring respectively ERdj4, ATF3 and BIP gene expression by qPCR. (TIFF) [file pone.0130635.s004.tiff]

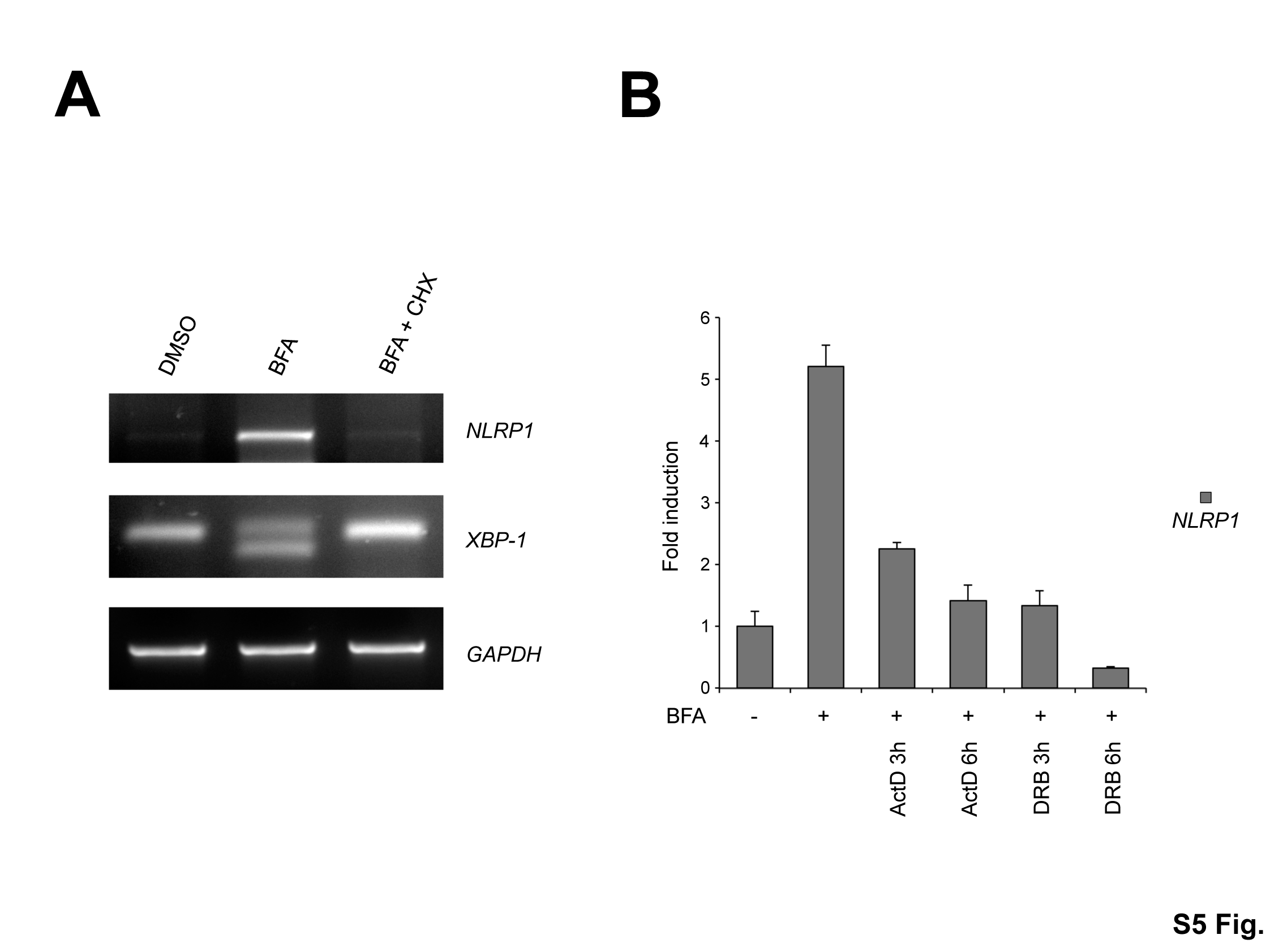

Supplement: S5 Fig — (A) HeLa cells were pre-incubated with CHX for one hour before overnight induction of ER stress with BFA. Cells were collected the following day and NLRP1, XBP-1 and GAPDH levels were measured by RT-PCR. (B) HeLa cells were incubated with DMSO or BFA for a total of 8 hours. Additionally, cells were exposed to transcriptional inhibitors ActD or DRB for the final 3 or 6 hours of BFA treatment. NLRP1 mRNA levels were evaluated by qPCR. (TIFF) [file pone.0130635.s005.tiff]

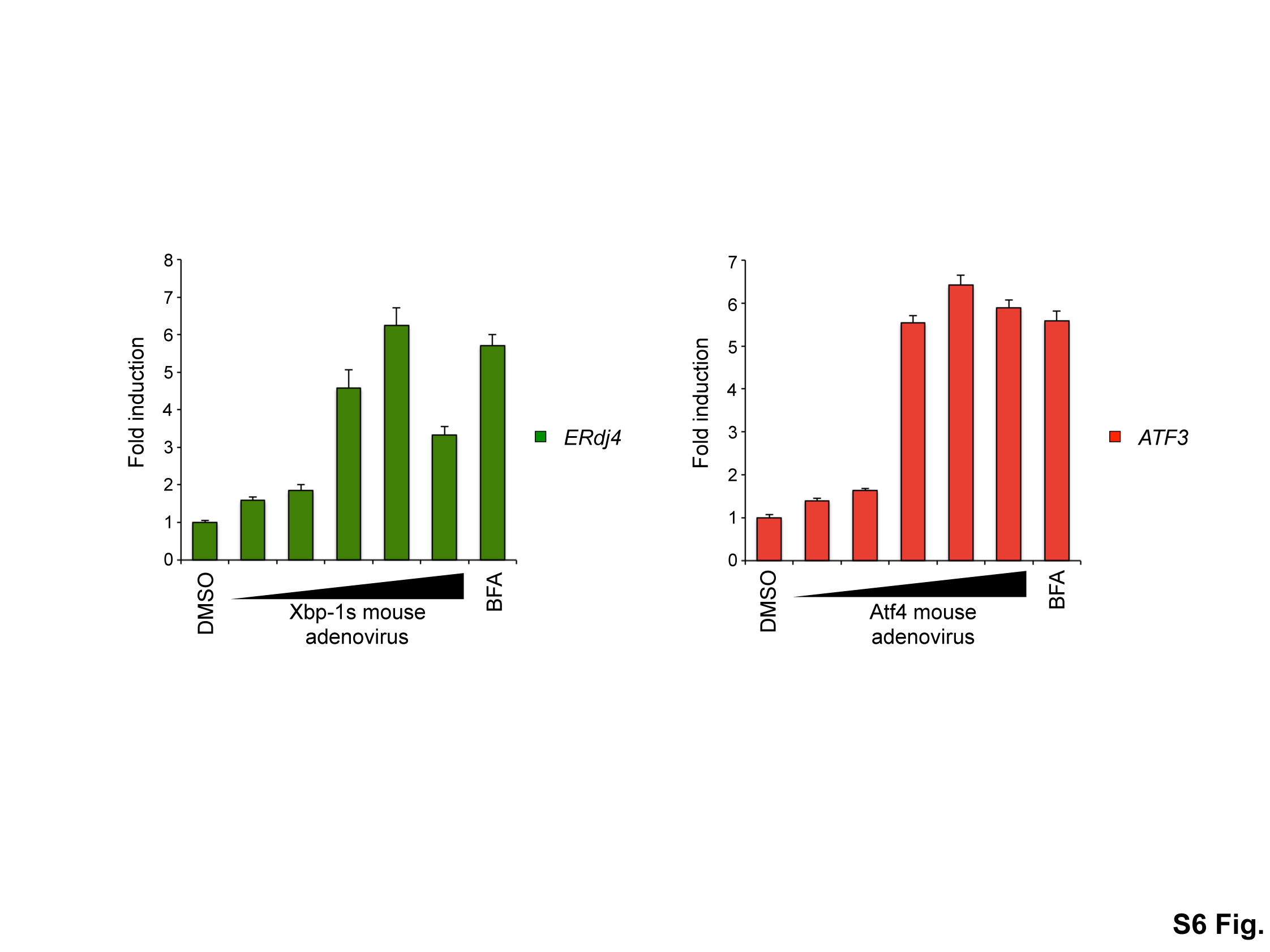

Supplement: S6 Fig — Functional validation of murine Xbp-1s and Atf4 adenovirus was performed by measuring gene expression of downstream targets by qPCR. Cells were treated as in Fig 3A. (TIFF) [file pone.0130635.s006.tiff]

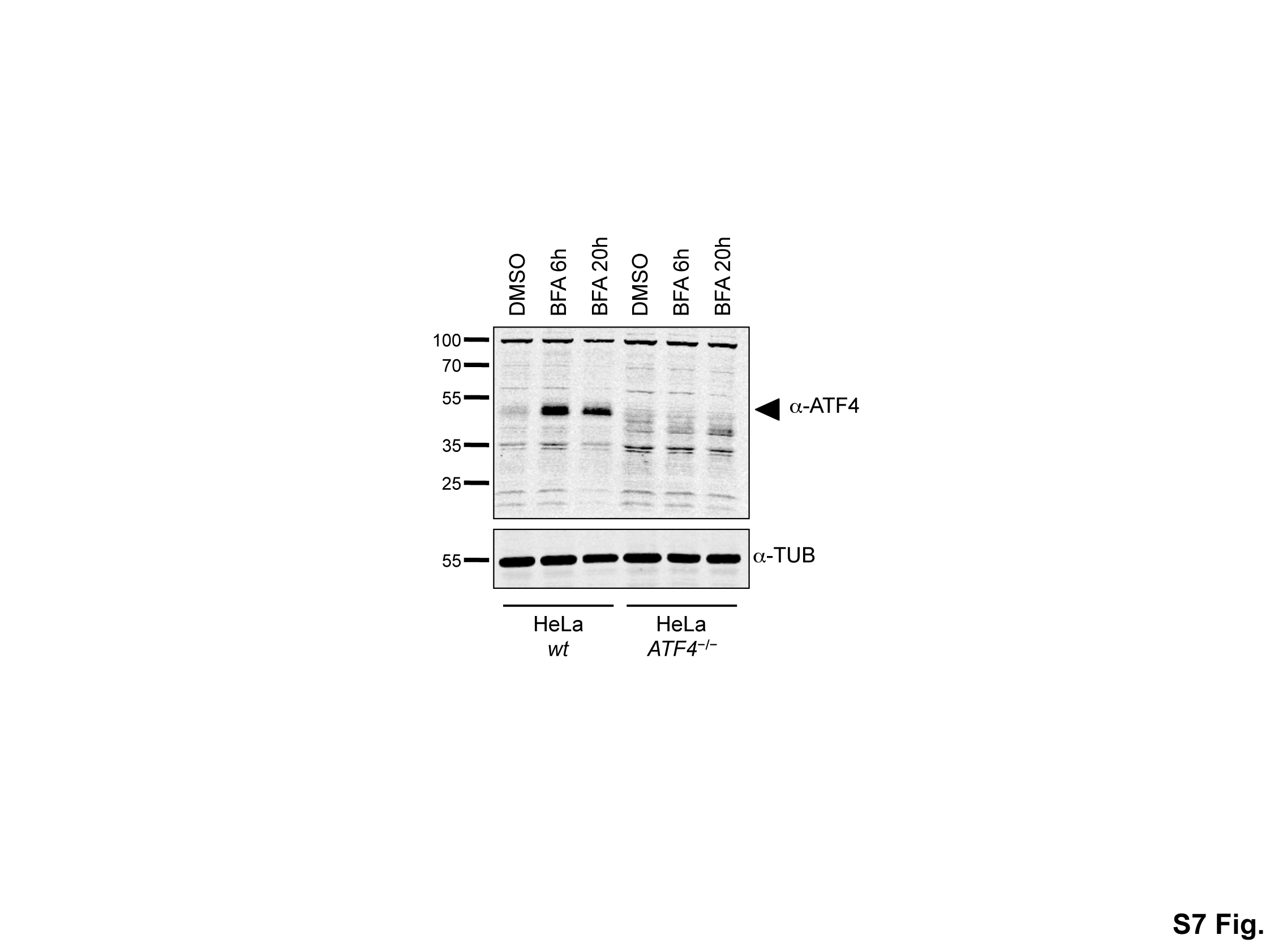

Supplement: S7 Fig — Cell lysates from wild-type or ATF4 −/− HeLa cells, untreated or treated with BFA for various times, were normalized for total protein content. Cell extracts were then subjected to SDS-PAGE/immunoblot analysis. Tubulin was detected as loading control. (TIFF) [file pone.0130635.s007.tiff]

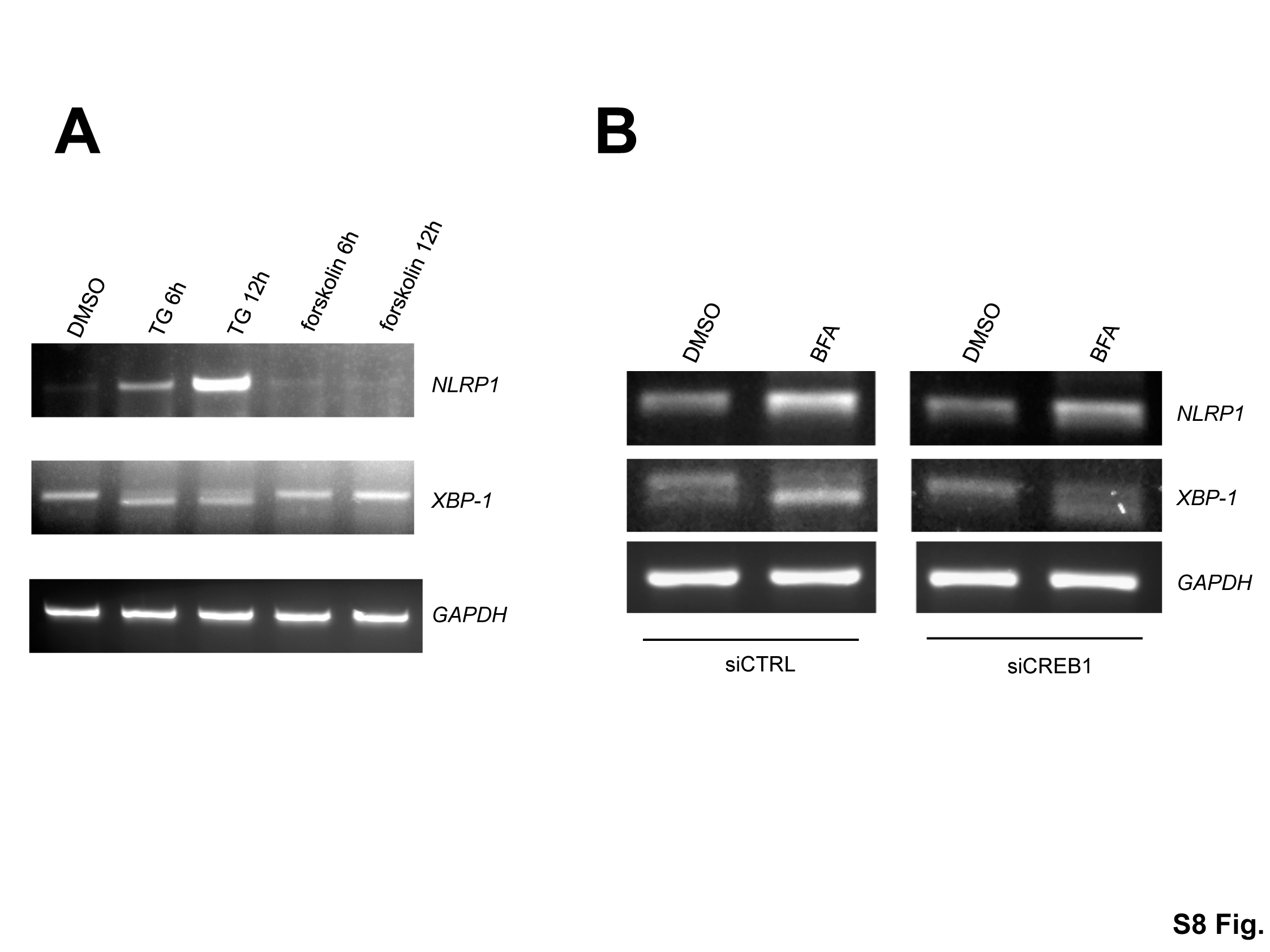

Supplement: S8 Fig — (A) HeLa cells were treated with either TG or Forskolin for the indicated times. NLRP1 and XBP-1 mRNA levels were evaluated by RT-PCR. (B) HCT116 cells were transfected with siRNA against CREB1 gene for 24 hours. Scrambled siRNA were used as control. Upon treatment with BFA for 16 hours, NLRP1 and XBP-1 mRNA levels were evaluated by RT-PCR. (TIFF) [file pone.0130635.s008.tiff]
